# Increased Extrasynaptic Glutamate Escape in Stochastically Shaped Probabilistic Synaptic Environment

**DOI:** 10.3390/biomedicines10102406

**Published:** 2022-09-26

**Authors:** Leonid P. Savtchenko, Dmitri A. Rusakov

**Affiliations:** UCL Queen Square Institute of Neurology, University College London, Queen Square, London WC1N 3BG, UK

**Keywords:** excitatory synapse, Monte Carlo model, glutamate spillover, glutamate uptake, synaptic cross-talk

## Abstract

Excitatory synapses in the brain are often surrounded by nanoscopic astroglial processes that express high-affinity glutamate transporters at a high surface density. This ensures that the bulk of glutamate leaving the synaptic cleft is taken up for its subsequent metabolic conversion and replenishment in neurons. Furthermore, variations in the astroglial coverage of synapses can thus determine to what extent glutamate released into the synaptic cleft could activate its receptors outside the cleft. The biophysical determinants of extrasynaptic glutamate actions are complex because they involve a competition between transporters and target receptors of glutamate in the tortuous space of synaptic environment. To understand key spatiotemporal relationships between the extrasynaptic landscapes of bound and free glutamate, we explored a detailed Monte Carlo model for its release, diffusion, and uptake. We implemented a novel representation of brain neuropil in silico as a space filled with randomly scattered, overlapping spheres (spheroids) of distributed size. The parameters of perisynaptic space, astroglial presence, and glutamate transport were constrained by the empirical data obtained for the ‘average’ environment of common cortical synapses. Our simulations provide a glimpse of the perisynaptic concentration landscapes of free and transporter-bound glutamate relationship, suggesting a significant tail of space-average free glutamate within 3 ms post-release.

## 1. Introduction

### 1.1. High-Affinity Astroglial Transporters Control Extrasynaptic Actions of Glutamate

One of the key principles underpinning our current understanding of the computational brain machinery is one-to-one ‘wired’ connectivity among individual neurons. The basic logic of neural-network learning algorithms, akin to the logic of electric circuits, has been developed based on this type of connectivity. However, thousands of glutamate molecules released from each synaptic vesicle at central excitatory synapses encounter only several dozens of their target receptors in the synaptic cleft before diffusing out into the perisynaptic extracellular environment. Because excitatory synapses in brain neuropil are only approximately 0.5 μm apart [1,2,3], glutamate escaping from one synapse can, in principle, activate its receptors at multiple neighbouring synaptic connections. To limit such activity, small excitatory synapses are often surrounded by ultrathin processes of astroglial cells, or astrocytes [4,5,6,7], enriched in high-affinity glutamate transporters expressed on the cell surface, in particular GLAST/GLT-1 type [8,9,10], among other signalling molecules [11,12]. By rapidly buffering escaping glutamate molecules, astroglial glutamate transporters constrain the extrasynaptic actions of glutamate. However, to what degree, and under what circumstances, such constraints would prevent activation of extrasynaptic glutamate receptors, and in particular glutamatergic inter-synaptic cross-talk, has remained a subject of debate, involving both theoretical and experimental exploration [13,14,15,16,17,18,19].

The importance of understanding these relationships also stems from the notion that the disruption of glutamate uptake has long been associated with pathological changes in the brain [20,21,22,23], whereas a pharmacological boost in glutamate uptake has recently been proposed to lessen age-related cognitive decline [24]. The issue has gained further prominence in view of the findings that relate drug addiction parameters to increased extrasynaptic glutamate actions in the nucleus accumbens [25,26,27], possibly through changes in the proximity of synapses and astroglial processes [28,29] which might eventually occur during heroin relapse [30].

### 1.2. Theoretical Models to Estimate Extrasynaptic Glutamate Escape

Monitoring the basic spatiotemporal features of single-vesicle glutamate release has recently become feasible thanks to the emergence of genetically encoded optical sensors [31,32]. However, resolving the events inside and outside the synaptic cleft is still heavily restricted by the diffraction limit of light microscopy, which is in the region of 200–250 nm for one-photon excitation and 300–400 nm for two-photon excitation regimes. The recently advanced live superresolution microscopy, such as STED imaging, could in principle improve this resolution limit, but only by 2–3-fold [33]. Because of these experimental limitations, simulation experiments and theoretical estimates—based on realistic biophysical models constrained by available experimental data—remain an important exploratory tool. Historically, attempts to realistically model glutamate release, extrasynaptic diffusion, uptake, and receptor activation progressed from multi-compartmental finite-difference models (e.g., [3,16,34]) to detailed Monte Carlo simulations, tracking individual molecules and their interactions (e.g., [15,35,36,37]). Generally, it is thought that 2000–3000 glutamate molecules are released by a single vesicle into the cleft, facing a few dozen synaptic AMPARs and several NMDARs. Once released, glutamate peaks, on the sub-millisecond scale, at a millimolar level, but only 15–20% of intra-cleft AMPARs are normally activated (e.g., [17]). Thus, neither AMPAR activation or their internalisation have an effect on glutamate escape.

With some computational models being considered advantageous or inferior to others in their specific aspects, the common limiting factor has been the implementation of reliable empirical constraints pertaining to the complex extracellular space geometry and the probabilistic presence of astroglial elements enriched in glutamate-binding transporters.

The majority of the previous nervous tissue models used regular arrays of cubes, other standard geometrical figures, or cell tessellations, to represent cell elements that determine the extracellular space geometry. In such models, the extracellular space would normally comprise a system of narrow clefts surrounded by flat parallel walls representing cell membranes. However, it has recently emerged that the extracellular space in the living brain neuropil occupies ~20% of the tissue volume, with the highly variable, randomly shaped inter-cellular (extracellular) voids sometimes reaching ~0.2 μm in width [38,39,40]. To account for this variability, we have recently introduced a model representation of neuropil cell elements in the shape of overlapping, randomly scattered spheres (spheroids) of varied size (see [41] and references therein). In this model, the extracellular cell fraction, the fractional presence of astroglia and their (glutamate uptake) binding capacity, and the size distribution of the spheroid elements can all be set in accordance with the empirical data. With this arrangement, the extracellular space shapes itself arbitrarily around cell elements, producing interconnected, concave as well as convex voids up to 100–200 nm in width. With this modelling approach, taking 10–20 space realisation runs helps generate data that represent an ‘average’ environment of common cortical synapses.

### 1.3. Glutamate Binding by Transporter-Enriched Astroglial Surfaces

In our previous simulations using a multi-spheroid model of synaptic environment [41], the binding between glutamate molecules and high-affinity astroglial transporters was assumed to occur when the diffusing molecule encounters the astroglia-attributed surface, with a probability between 0.13 and 0.3 reflecting the volume fraction occupied by astroglia. However, because both simulated and real glutamate molecules diffuse with a translational step of only a few nanometres, all spheroid surfaces would thus be seen in the model as glutamate-binding surfaces within only a few diffusion-step iterations. The resulting simulation outcome [41] would thus represent the case of synapses surrounded exclusively by astroglial processes, with an unlimited binding capacity. This case might be representative of the excitatory synapses surrounded by Bergmann glia in the cerebellum [42], calyceal synapses [43], or synaptic glomeruli [44], but could be less relevant for other brain regions, such as hippocampal neuropil. Thus, the previous results [41] should be interpreted as follows: the extent of spatial escape routes in the synaptic environment have little effect on glutamate escape as long as the synapse is surrounded by the glutamate transporter-expressing (astroglial) processes only.

To extend the physiological relevance of our model, in the present study, we make a more realistic case in which an experiment-established proportion of cell membranes related to astrocytes is represented by spheroid structures that are designated accordingly, from the start. Thus, simulated cell membranes that surround synapses represent neuronal and astroglial elements in the proportion observed empirically. Another important improvement was an introduction of the stochastic-binding time constant Ψ, which combines the effects of affinity and surface density for astroglial glutamate transporters.

## 2. Methods

### 2.1. Stochastic Model of Neuropil Geometry

Monte Carlo simulations were carried out over the 4 μm wide cube arena, normally with 1000 Brownian particles representing glutamate molecules released instantaneously into the synaptic cleft (120 nm wide 20 nm tall disk) at the centre. Our previous work introduced the model of nerve tissue based on the stochastically generated geometry of neuronal and astroglial elements, which thus formed the extracellular space architecture [41]. In brief, all the space outside the synaptic cleft was occupied by randomly scattered, overlapping sphere shapes, with the closest approach of any sphere surface to the cleft restricted to ~10 nm. To fill the volume with the overlapping spheres of distributed size, we generated random 3D coordinates of sphere centroids in the simulation arena and a random radius value for each sphere. The sphere radius was distributed uniformly at 50 and 300 nm to reflect (approximately) the appearance and size of neuronal and astroglial elements seen in 3D-EM reconstructions of the synaptic neuropil [36,45]. This procedure was carried out anew for every new simulation run. The tissue volume fraction α occupied by the extracellular space varied between 0.1 and 0.3 [46,47,48]. The value of α was validated, in each simulation run, by (i) using an evenly random distribution of ‘test points’ over the entirety of the 3D arena, and (ii) calculating the fraction of the ‘test points’ falling inside spheres [41]. The underlying Monte Carlo algorithms, computational approaches, and methods employed here have been detailed and validated previously (see [15,41] and references therein). The diffusion coefficient in a free medium was set at *D* = 0.5 μm^2^ /ms in accordance with the in situ measurements of extracellular diffusivity in brain slices using time-resolved fluorescence anisotropy imaging (see [41] and references therein).

### 2.2. Interaction between Diffusing Molecules and Simulated Cell Surfaces

The mode of interaction between diffusing particles (glutamate molecules) and the surface of spheroids depended on whether the latter represented a designated neuronal or astroglial membrane. The spheres representing astroglia comprised ~10% of the simulated tissue volume, in accordance with the quantitative EM data for hippocampal neuropil (see [49] and references therein). In the case of ‘neuronal’ spheroids, the interaction with glutamate was simulated as an elastic (mirror reflection) collision. In contrast, the surface of ‘astroglial’ spheroids was assumed to express high-affinity glutamate transporters. In that case, for each diffusing particle, the interaction was simulated as a stochastic binding event that may occur with probability *P*. For each particle, *P* was a function of time *t* elapsed from the particle’s first collision with the ‘astroglial’ spheroid, in accordance with the classical lifetime expression for first-order reactions, *P* = 1−exp(*t*Ψ^−^^1^). Here, Ψ is the time constant (free parameter) that determines how fast-approaching or imminent the forthcoming binding event is when the particle remains in the vicinity of the astroglial surface. Parameter Ψ thus combined, in a single quantity, the effects of transporter binding affinity, the binding-site (transporter) cell surface density, and binding surface proximity to the diffusing particle. In practice, for each particle, computing *P* continued as long as the particle was within 5 nm of the ‘astroglial’ spheroid; it was reset to zero once the particle departed from the ‘astroglial’ surface by >5 nm. We tested that increasing the cut-off distance above 5 nm had no detectable effect on *P*.

Overall, our test runs were restricted to a 4–5 ms period, a characteristic time for diffusion equilibration over the chosen simulation arena. Therefore, once bound, the glutamate particles remained bound to astroglial spheroids for the simulated time interval, as the characteristic time of glutamate unbinding for the main glial glutamate transporter GLT-1 is tens of milliseconds [50,51].

Monte Carlo simulations were run using two computing environments. The first environment was a UCL Myriad cluster: processors per node, Intel(R) Xeon(R) Gold 6240 CPU @ 2.60 GHz; cores per node 36 + 4 A100 GPUs; RAM per node 192 GB, tmpfs 1500 G, total 6 nodes. The second environment was cloud computing with Amazon AWS: t4g.medium, memory 4 GB. Parallelisation and optimisation of the algorithms and program codes were implemented by AMC Bridge LLC (Waltham, MA, USA), with internet security assistance from Cybecurio Ltd. (Berkhamsted, UK).

## 3. Results

### 3.1. Glutamate Release, Diffusion, and Transporter Binding

In the first set of simulation experiments, the system parameters were set to match the ‘average’ geometry of synaptic neuropil within one of the most studied regions of the rodent brain—area CA1 of the hippocampus. Our preliminary exploration of time constant Ψ indicated that its value of ~1 ms would best correspond to the spatial profile of synaptically released glutamate bound to its optical sensor iGluSnFR in this brain region [32].

**Figure 1:**
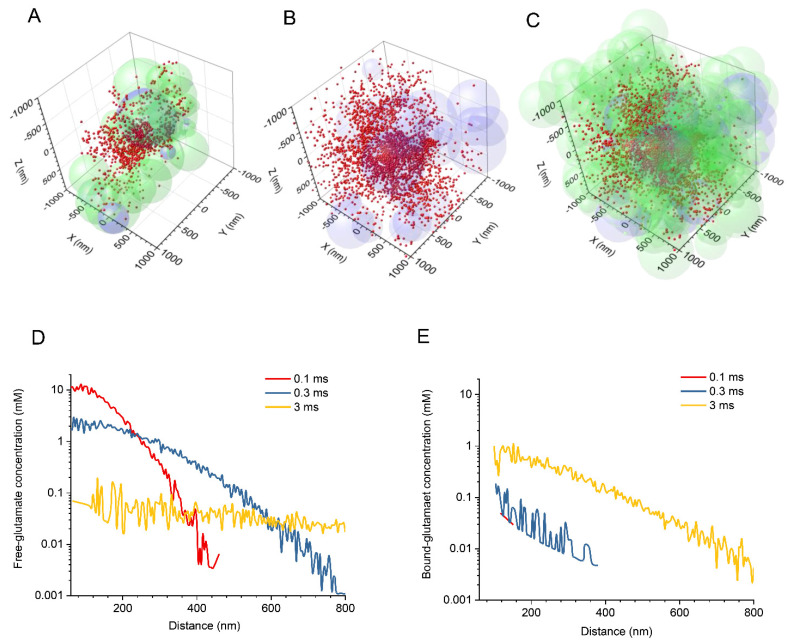
Exploring extrasynaptic glutamate escape in silico with a stochastic model of synaptic neuropil and Monte Carlo simulations. (**A**–**C**) Diagrams illustrating a snapshot of simulated glutamate particles (red dots) escaping the synaptic cleft at 1 ms post-release. The extracellular space is filled with overlapping spheres representing neuronal (light green) and astroglial (light magenta) structures. For clarity, the same data set is shown within a restricted volume slab (**A**), with astroglial elements only (**B**), and with all the components (**C**). The diagram shows a 2 μm wide fragment of the 4 μm wide simulation arena. Key model parameters: α = 0.2, free-diffusion *D* = 0.5 μm^2^ /ms, Ψ = 1 ms, astroglia fraction ~10%; number of molecules, 1000, average of *n* = 10 trials. (**D**) Simulated spatial profiles of the free glutamate concentrations, with respect to the cleft boundary (60 nm), at three time points post-release as indicated by colour code. (**E**) The same as in D but for the concentration of transporter-bound glutamate. Note that the distribution starts, on average, at ~20 nm outside the synaptic cleft.

The snapshots of simulation at 1 ms post-release displayed a characteristic cloud of glutamate molecules that diffuse in the extracellular space while binding to ‘astroglial’ surfaces (Figure 1A–C). These simulations were used to estimate the rapidly evolving extracellular concentration profiles of free-diffusing (Figure 1D) and transporter-bound glutamate (Figure 1E) in the extracellular space. The free-bound relationship for glutamate changes dramatically on the millisecond timescale. In the vicinity of the synapse, it drops from 100–300 at 0.1–0.3 ms, to 0.1 at 3 ms post-release (Figure 1D,E). At 250–300 nm from the release site, the change is from 200 at 0.3 ms to 0.15–0.2 at 3 ms. Perhaps surprisingly, the simulations predict a >10 μM ‘tail’ of free glutamate extending, on average, across the simulation arena as time progresses (Figure 1D; see Discussion).

### 3.2. Exploring Free Parameters

**Figure 2:**
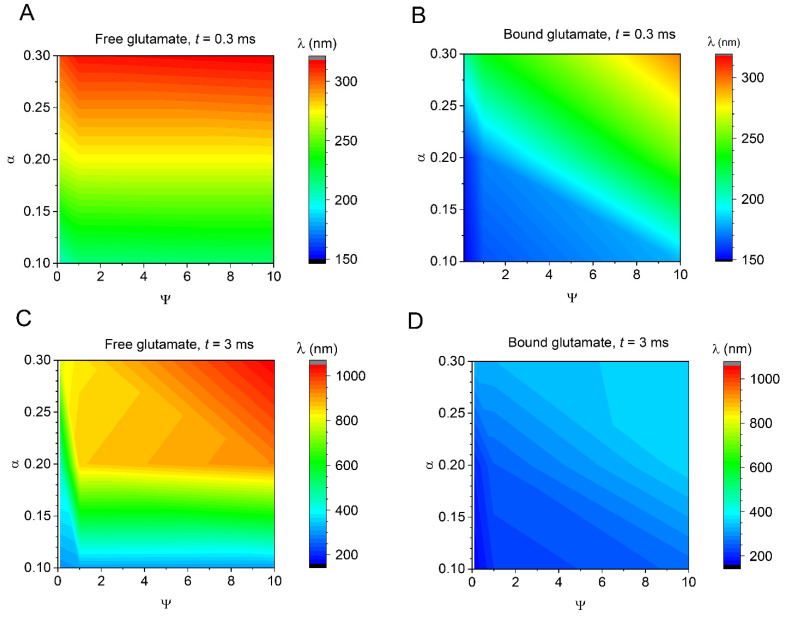
The influence of binding efficacy and extracellular space fraction on the glutamate escape profile. (**A**) Length constant λ (distance at 1/*e* top value near at the synaptic cleft boundary) of the extracellular free-glutamate concentration profile, false-colour coded (scale bar shown), in relation to the range of Ψ and α, as indicated; time point, 0.3 ms post-release. (**B**) Same as (**A**), but for transporter-bound glutamate. (**C**) Same as (**A**), but for the time point of 3 ms post-release. (**D**) Same as (**B**), but for the time point of 3 ms post-release.

To better understand how the (assumed or estimated) system parameters influence the outcome of our simulations, we carried out a series of tests in which we varied the values of Ψ and α over a physiologically plausible range. For the sake of brevity, the outcome of these simulations was represented by the characteristic length constant λ (a spatial decay by *e* times) of the glutamate concentration with respect to the external boundary of the cleft. The results illustrate the relationship between Ψ, α, and λ at the two time points post-release, 0.3 and 3 ms (Figure 2). As expected, the data suggest that increasing Ψ or α, hence weakening glutamate transporter buffering capacity or expanding the escape routes, would extend the spatial spread of bound glutamate as the time progresses (Figure 2B,D). As for the free glutamate, a similar trend appears pronounced across the tested range of α, but only for the smallest tested Ψ values, representing the greatest transporter efficacy (Figure 2A,C). Again, a somewhat surprising result here is that the tail of free glutamate remains relatively far reaching across the tested range of parameters (Figure 2A,C; see Discussion).

## 4. Discussion

In our previous work, we introduced a computational model of synaptic neuropil in silico, in which the space is represented by randomly scattered, stochastically sized overlapping spheres [41]. In this model, each simulation run generates different space geometry while keeping all its statistical parameters unchanged. Unlike the traditional model representations of brain tissue, which normally involve an array of regular geometric shapes, the ‘random-spheroid’ model incorporates statistical uncertainty, which is characteristic of real tissue. The logic of stochastic representation also applies to the distribution of astroglial as opposed to neuronal elements in this model, which reflects such variability in experimental observations.

With this modelling approach, our previous attempt to assess extrasynaptic glutamate escape, diffusion, and transporter binding assumed unlimited efficacy of high-affinity transporters expressed on sphere surfaces [41]. Under that assumption, the majority of glutamate molecules escaping the cleft appeared bound by high-affinity transporters in close proximity of the synapse, leaving only a small fraction to diffuse further [41]. As mentioned above, the set of model parameters in that study was likely to represent synapses surrounded almost entirely by astroglia, such as the case of Bergmann glia in the cerebellum [42], calyceal synapses [43], or synaptic glomeruli [44]. In the present work, the occurrence of designated astroglial or neuronal structures near the synapse was dictated by the experimentally established proportion of such structures as seen in the neuropil more commonly. Again, every realisation of the model would stochastically generate this structural arrangement anew, thus reflecting the variability (hence the uncertainty) of a synaptic environment observed in real life.

In this context, our experiments involving the high-resolution imaging of glutamate escape with an optical glutamate sensor suggested that the spatial profile of extrasynaptic glutamate bound by the optical sensor, transporter, or receptor could indeed be much wider [32] than predicted by our previous model [41]. We therefore introduced a free ‘operator’ parameter Ψ that synthetically represents the efficacy of glutamate binding to astroglia in the model, so that the spatial profile of bound glutamate is compatible with the experimental observations. With what appears to be a realistic range of Ψ, our simulations here predict, perhaps surprisingly, a significant spatial spread of both bound and free glutamate outside the synaptic cleft post-release.

With the adopted glutamate diffusivity, the concentration profile generated by a quasi-instantaneous point source becomes fairly flat at 3 ms post-release on the micron scale (Figure 1D), suggesting the average concentration of ~10 μM at this point. However, the average value does not reflect local variations: the presence of glutamate binding sites (transporters or receptors) reduces its local concentration rapidly, while in the binding-free areas, free glutamate molecules could ‘bounce around’ for longer. We expect therefore that the average glutamate level will also drop rapidly to its sub-micromolar values beyond the millisecond scale, although such long-term simulations are currently technically prohibitive. One consequence of this result is that the glutamate receptors that have no nearby high-affinity transporters could be exposed, albeit very briefly, to ~10 μM levels of glutamate. Whether such exposure on the millisecond time scale could activate a significant proportion of such receptors (and thus, for instance, desensitise them) is an intriguing question. For comparison, AMPA receptors in the synaptic cleft are exposed to millimolar peak concentrations of glutamate at the sub-millisecond time scale, yet only 20–30% of them get activated and hence are subject to desensitisation (e.g., [17]).

Experimental observations in brain slices have long reported that, in certain conditions, escape of glutamate from excitatory synapses could lead to functional cross-talk between neighbouring synapses [13,14,15,16]. If common and significant, such cross-talk would challenge the traditional neural network theories based on the assumption of the one-to-one, wired information transfer among brain cells. However, to what extent this volume-transmitted mode of communication occurs in physiological conditions in the intact brain remains to be ascertained, and the present simulations help dissect key factors contributing to such phenomena. Equally important would be the knowledge about how far glutamate can extend its extrasynaptic actions when the brain develops pathological changes [22,23] and whether pharmacological or genetic manipulation of glutamate uptake mechanisms could correct the malfunction of brain networks associated, for instance, with drug addiction progress such as heroin relapse [28,29,30].

## Author Contributions

L.P.S. designed and carried computer simulations; D.A.R. narrated the study, provided the concept, and wrote the manuscript. All authors have read and agreed to the published version of the manuscript.

## Data Availability

All the raw data generated in this article are freely available on request.
